# Ribotyping for Accurate Identification of Infectious Bacteria in Animal‐Derived Foods and Laboratory Samples: Implications for Human Health

**DOI:** 10.1155/jotm/2873071

**Published:** 2026-09-07

**Authors:** Zahra Pourramezan Seyedi, Baharak Mirzavand, Majid Taati Moghadam, Mohammad Taghi Ashoobi, Jamile Nowsani

**Affiliations:** ^1^ Department of Biology, Faculty of Basic Sciences, Science and Research Branch, Islamic Azad University, Tehran, Iran, azad.ac.ir; ^2^ Department of Pathobiology, Faculty of Veterinary Medicine, Shahid Chamran University of Ahvaz, Ahvaz, Iran, scu.ac.ir; ^3^ Department of Microbiology, Virology and Microbial Toxins, School of Medicine, Guilan University of Medical Sciences, Rasht, Iran, gums.ac.ir; ^4^ Otorhinolaryngology Research Center, School of Medicine, Guilan University of Medical Sciences, Rasht, Iran, gums.ac.ir; ^5^ Razi Hospital, Guilan University of Medical Sciences, Rasht, Iran, gums.ac.ir; ^6^ Department of Vascular Surgery, Razi Clinical Research Development Unit, Razi Hospital, Guilan University of Medical Sciences, Rasht, Iran, gums.ac.ir; ^7^ Department of Microbiology, Karaj Branch, Islamic Azad University, Karaj, Iran, azad.ac.ir

**Keywords:** animal-derived foods, antibiotic resistance, foodborne pathogens, molecular typing, ribotyping

## Abstract

Ribotyping is a molecular typing approach based on ribosomal RNA (rRNA) gene sequences for the identification and characterization of bacterial strains. This review aims to evaluate the effectiveness of ribotyping in the identification of infectious bacteria in animal‐derived foods and veterinary samples. A narrative literature review was conducted using major scientific databases, including PubMed, Scopus, Google Scholar, and Web of Science, covering studies published to 2025. Relevant articles were selected based on their focus on ribotyping methodologies (e.g., RFLP‐, PCR‐, and automated ribotyping) and their applications in food safety, veterinary microbiology, and zoonotic disease investigations. The findings indicate that ribotyping has been widely applied for epidemiological investigations, source tracking, and characterization of foodborne and zoonotic pathogens. These approaches have contributed to understanding bacterial diversity and monitoring antibiotic resistance patterns in animal populations and related food products. However, compared with high‐resolution molecular techniques such as whole genome sequencing (WGS), ribotyping demonstrates lower discriminatory power and limited resolution for fine‐scale epidemiological analysis. Despite these limitations, ribotyping remains a useful, accessible, and cost‐effective tool in certain laboratory and surveillance settings, particularly where advanced genomic technologies are not readily available. Overall, integrating ribotyping with newer genomic approaches can enhance the monitoring and control of infectious bacteria, thereby supporting animal health, food safety, and public health outcomes.

## 1. Introduction

Ribotyping is a molecular typing method based on the analysis of ribosomal RNA (rRNA) gene regions for the identification and classification of bacteria. This approach exploits sequence variability within conserved rRNA loci, enabling differentiation among bacterial taxa while maintaining analytical stability across diverse species. By generating characteristic banding patterns from ribosomal DNA (rDNA), ribotyping provides reproducible molecular fingerprints that can be used for strain‐level discrimination in epidemiological investigations [1–3]. Since its introduction in the late 1980s and early 1990s, ribotyping has been applied in clinical microbiology, food safety, and veterinary research as a tool for bacterial characterization. Early applications demonstrated its utility in distinguishing bacterial strains and tracing sources of infection in both clinical and foodborne outbreak settings. In particular, ribotyping has been used to differentiate closely related strains of pathogens such as *Salmonella* spp. and *Clostridioides difficile*, supporting epidemiological analyses across human and animal populations [4, 5]. In veterinary and food microbiology contexts, ribotyping has been employed to identify infectious bacteria associated with livestock, companion animals, and animal‐derived food products. Its application enables the comparison of isolates from different sources, thereby supporting source attribution and the investigation of transmission pathways. Although ribotyping has been widely used, its application in animal‐derived food systems remains underexplored compared with genome‐based approaches, particularly in studies requiring high‐resolution discrimination [4]. Infectious bacteria associated with animal‐derived foods represent a critical interface between animal health and human disease. Pathogens such as *Escherichia coli*, *Salmonella* spp., and *Campylobacter* spp. are frequently implicated in foodborne infections and are commonly linked to products of animal origin. Transmission routes are diverse and include direct contact with infected animals, consumption of contaminated food products, and exposure to contaminated environments. These transmission pathways contribute to the global burden of foodborne disease and pose ongoing challenges for public health systems [4, 6–9]. The increasing prevalence of antimicrobial resistance (AMR) among bacterial pathogens further complicates the management of infections originating from animal sources. Resistant strains may emerge through selective pressure in livestock production systems and can be transmitted to humans through the food chain or direct contact. This highlights the importance of accurate identification and characterization of infectious agents to support effective surveillance, control measures, and risk assessment strategies [10–16]. Timely identification of bacterial pathogens remains essential for outbreak investigation and infection control. Molecular typing methods such as ribotyping contribute to this process by enabling differentiation among bacterial strains and facilitating epidemiological linkage between isolates. Although ribotyping does not provide genome‐wide information, its methodological simplicity, reproducibility, and relatively low technical requirements make it a practical option in certain laboratory settings, particularly where access to advanced sequencing technologies is limited [6, 17]. This review evaluates the application of ribotyping for the identification of infectious bacteria in animal‐derived foods and laboratory samples. It examines the methodological basis of ribotyping, its practical applications in veterinary and food microbiology, and its relevance within the broader context of bacterial typing approaches used in public health and epidemiological investigations.

## 2. What Is Ribotyping?

Ribotyping is a molecular typing method based on the analysis of rRNA gene regions to differentiate bacterial species and, in some cases, strains. This approach exploits the conserved nature of rRNA genes alongside variations in the number, distribution, and flanking regions of ribosomal operons within bacterial genomes. As a result, digestion patterns generated from these regions can be used to produce strain‐specific fingerprints. Classical ribotyping is primarily based on restriction fragment length polymorphism (RFLP) analysis of genomic DNA, followed by Southern blot hybridization using labeled rRNA gene probes. The resulting banding patterns (ribotypes) allow comparison among isolates and have been widely applied in epidemiological investigations, clinical microbiology, and food safety studies [18–20]. Ribotyping has been reported to provide acceptable levels of specificity and sensitivity in clinical microbiology applications; however, its performance may vary depending on the bacterial species, methodological approach, and experimental conditions. The specificity of ribotyping is largely attributed to the analysis of conserved yet polymorphic regions within rRNA genes, which can enable differentiation among bacterial strains in certain contexts. Nevertheless, the extent of discrimination may be limited when closely related strains share highly similar ribosomal patterns. In terms of sensitivity, ribotyping can detect bacterial contamination when sufficient DNA is present; however, its detection capacity is influenced by factors such as sample complexity, DNA quality, and methodological workflow. While several studies have demonstrated its applicability in identifying pathogenic bacteria in food and animal samples, its performance is not universally consistent across all microbial groups or epidemiological settings. Therefore, ribotyping is best considered a useful and context‐dependent tool rather than a universally high‐resolution method for bacterial identification [7, 17, 21, 22]. In veterinary and food microbiology, ribotyping has been applied for the identification and tracking of infectious bacteria, supporting outbreak investigations and source attribution. Its relative technical simplicity and reproducibility have contributed to its continued use in routine laboratory settings, particularly where access to advanced genomic technologies is limited [7, 17, 21, 22].

It is important to clarify that ribotyping is an umbrella term that includes different methodological approaches based on rDNA variation analysis. Classical ribotyping relies on restriction enzyme digestion of genomic DNA followed by Southern blot hybridization, whereas polymerase chain reaction (PCR)–based approaches amplify ribosomal gene regions prior to downstream analysis. In contrast, 16S rRNA gene sequencing is primarily used for taxonomic identification and phylogenetic analysis rather than strain‐level typing [23, 24].

### 2.1. History of Ribotyping

Ribotyping was introduced in the late 1980s as a molecular approach for characterizing bacterial strains based on variation in rDNA. Early applications focused on organisms such as *Escherichia coli*, where restriction analysis of rDNA enabled differentiation among strains associated with foodborne outbreaks [24, 25]. This method represented a significant advancement over phenotypic typing techniques, offering improved reproducibility and genetic resolution. Over time, ribotyping became widely adopted in epidemiological studies, clinical diagnostics, and food microbiology, particularly for tracking bacterial transmission and contamination sources [26, 27].

### 2.2. Types of Ribotyping Methods

Various methods exist for performing ribotyping, with the most common being RFLP analysis of rDNA. This approach involves digesting rDNA with specific restriction enzymes, followed by gel electrophoresis to separate the resulting fragments based on size. The patterns generated are then compared against established databases or reference strains to identify the bacterial species or strain. In recent years, advancements such as PCR‐based techniques have been introduced, allowing for more rapid and specific amplification of rRNA genes prior to analysis [5, 28]. Additionally, whole genome sequencing (WGS) is emerging as an alternative to traditional ribotyping, providing comprehensive genetic information that can complement or enhance ribotyping results [5]. Ribotyping encompasses several methodologies that vary in complexity and specificity. Classical ribotyping, PCR‐ribotyping, and 16S rRNA gene sequencing represent distinct molecular approaches that should be clearly differentiated based on their methodological principles and applications. Conventional ribotyping is based on restriction enzyme digestion of genomic DNA followed by Southern blot hybridization. PCR‐ribotyping involves amplification of ribosomal intergenic spacer regions prior to fragment analysis and is widely used for organisms such as *Clostridioides difficile*. In contrast, 16S rRNA gene sequencing is primarily used for bacterial identification and phylogenetic classification rather than strain differentiation.

#### 2.2.1. Conventional Ribotyping

Conventional ribotyping is a molecular technique based on RFLP. In this method, total genomic DNA is first digested using restriction endonucleases [7]. The resulting fragments are then separated by electrophoresis, transferred onto a membrane via Southern blotting, and subsequently hybridized with a radiolabeled probe targeting ribosomal operons. With the advancement of ribosomal gene sequencing and DNA analysis software, it has become feasible to select restriction enzymes that cleave once within the 16S rRNA gene and once within the 23S rRNA gene. This allows for the identification of sequence polymorphisms within ribosomal operons as well as in adjacent upstream and downstream flanking regions [7]. The DNA used in this process is typically extracted from a pure bacterial culture originating from a single colony. Following extraction, the DNA undergoes restriction digestion to produce fragments of defined sizes. These fragments are separated electrophoretically and transferred to a membrane. Hybridization is then carried out using labeled probes, which may be radioactive, fluorescent, or chemiluminescent. For preparing species‐ or genus‐specific probes, the entire 16S–23S–5S rRNA operon is usually amplified by PCR. The amplified product is then used to generate labeled probes through random priming with the Klenow fragment of DNA polymerase I. Alternatively, probes can be prepared by cloning specific regions of the operon (such as 16S or 23S) or by synthesizing cDNA from rRNA templates using reverse transcriptase [7]. Chemically synthesized oligonucleotides containing taxon‐specific signature sequences may also serve as probes. After electrophoresis and hybridization, the resulting banding patterns corresponding to rRNA genes (ribotypes) are recorded, digitized, and analyzed either manually or with specialized software and subsequently stored in databases. Differences in ribotype patterns among bacterial isolates arise from variations in both the position and intensity of bands, which reflect sequence diversity. These variations are useful for bacterial identification and classification. When the full *rrn* operon is used as the probe, the resulting pattern is referred to as a ribotype, whereas the use of only the 16S rRNA gene produces what is known as a 16S ribotype. The observed polymorphisms are indicative of the conserved nature of rRNA operon sequences alongside variability in the surrounding chromosomal regions. In some cases, to improve strain discrimination, multiple restriction enzymes may be applied simultaneously, increasing the level of detectable polymorphism in restriction fragment lengths [7].

#### 2.2.2. PCR‐Ribotyping

Previous PCR‐based studies have identified genetic variants of microbes through sequence analysis with cloning, which is labor‐intensive, time‐consuming, and costly. To assess zoonotic infections in numerous samples from diverse regions, more precise, efficient, and cost‐effective methods are needed. PCR‐RFLP is a widely used tool for analyzing the molecular characteristics of pathogens. It serves various purposes, such as species differentiation, pathogenicity prediction, and genotypic analysis [29]. In RFLP‐ribotyping, genes encoding rRNA are targeted using a labeled probe that contains either 16S, 23S, or both 16S and 23S ribosomal cDNA. Prior to hybridization, the DNA is digested with enzymes such as BamHI, EcoRV, PvuII, or NarI and then transferred onto a membrane using Southern blotting. The resulting hybridization patterns, or ribotypes, produced by the probe binding to different DNA fragments allow for differentiation between bacterial species [30]. To execute the RFLP‐ribotyping method, several steps are carried out sequentially. Obtain bacterial isolates from clinical samples, food products, or laboratory animals. Use a suitable DNA extraction kit or protocol to isolate genomic DNA from the bacterial cells. Amplify specific regions of the rRNA genes (commonly 16S rRNA) using PCR with specific primers designed for these regions. Treat the amplified PCR products with one or more restriction enzymes that cut the DNA at specific sequences. This step generates fragments of varying lengths based on the genetic makeup of the bacteria. Separate the digested fragments using agarose gel electrophoresis. The gel is then stained with a DNA‐binding dye (e.g., ethidium bromide) to visualize the bands. Capture images of the gel and analyze the banding patterns using software or manual comparison against known ribotype patterns stored in databases. The resulting banding patterns are compared to reference profiles from established databases or previous studies to identify the bacterial strain. Each distinct pattern corresponds to a specific ribotype, allowing for differentiation between closely related strains [24, 31–34].

#### 2.2.3. Automated Ribotyping

The RiboPrinter is a fully automated system for identification and subtyping that integrates the molecular steps discussed into a single instrument, enhancing speed and reliability. It provides standardized results that are comparable across laboratories. Like manual ribotyping, automated ribotyping offers high typeability, meaning it can subtype nearly all populations of a microorganism. Most studies report 100% typeability, although some exceptions exist, such as a study that reported 98.7% typeability for *Campylobacter* spp. isolates. Automated ribotyping is efficient, allowing analysis to be completed in less than 8 h, enabling the RiboPrinter to process up to 32 fresh cultures per day. As with all genotyping methods, automated ribotyping relies on effective sampling and culturing procedures prior to analysis [26, 35]. The process involves several key steps, from bacterial sampling to the interpretation of results. Overnight bacterial cultures are grown on appropriate agar plates, such as brain heart infusion agar. Cells are picked from the agar plates and suspended in a sample buffer. The bacterial cells are inactivated using a heat kill step to ensure safety and facilitate DNA extraction. Lytic enzymes are applied to break down the cell walls and release the genomic DNA. The extracted DNA is digested with specific restriction enzymes, such as EcoRI or PvuII, which cut the DNA at particular sequences. This step is crucial for generating fragments that will be used for analysis. The digested DNA fragments are separated by size using gel electrophoresis. This separation allows for the visualization of distinct bands corresponding to different DNA fragments. The separated DNA fragments are transferred onto a membrane using Southern blotting techniques. This step prepares the fragments for hybridization with a probe [3, 26, 36–38]. A radiolabeled ribosomal operon probe is hybridized to the transferred DNA fragments on the membrane. The probe binds specifically to rDNA sequences, allowing for the visualization of relevant bands. The hybridized bands are visualized using autoradiography or chemiluminescent detection systems. A digitizing camera captures the light emission as image data, from which the system extracts a RiboPrint pattern. The digitized image is analyzed using software that characterizes and identifies the ribotype patterns. The system is equipped with software that analyzes the output, using molecular weight standards as a reference to normalize it. The results can be statistically evaluated and compared to a built‐in strain‐pattern database. If a pattern does not match any existing patterns in the database, a new ribotype is created, saved, and retained for future reference, thereby enhancing the system’s recognition capabilities over time. Nonetheless, strain differentiation is still possible even when an isolate is not in the identification database by comparing the corresponding band patterns. The system requires very little maintenance because all necessary materials, including reagents and even rinsing water, are provided in ready‐to‐use, prepackaged components [3, 26, 36–38].

## 3. The Challenge of Infectious Bacteria in Animal‐Based Foods and Samples

**FIGURE 1:**
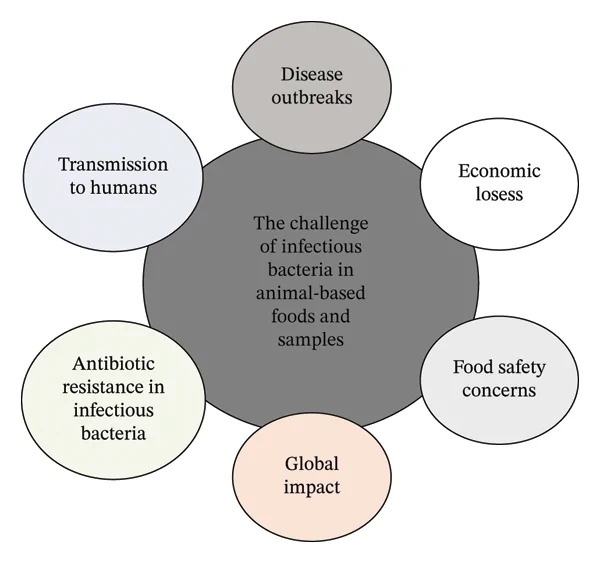
Factors that complicate the identification of infectious bacteria in food and animal samples.

Infectious bacteria associated with animals, animal‐derived foods, and related clinical samples represent a significant challenge for public health, veterinary medicine, and the agricultural sector. The consequences of these infections extend beyond animal health and directly impact food safety, economic stability, and human health. This section examines the major challenges posed by infectious bacteria, including their health and economic burden, AMR, transmission pathways to humans, and global disparities in management and control strategies (Figure 1) [39–41].

### 3.1. Health and Economic Impacts of Infectious Bacteria Relevant to Bacterial Identification

Infectious bacterial diseases in animals are associated with a wide range of clinical outcomes that affect livestock productivity, animal welfare, and food production systems. These infections frequently result in high morbidity and mortality rates, particularly during outbreaks. Several well‐documented diseases illustrate these impacts. Bovine tuberculosis, caused by *Mycobacterium bovis*, primarily affects cattle but is also zoonotic, leading to chronic respiratory disease and substantial economic losses due to decreased milk production and culling of infected animals [42]. Avian cholera, caused by *Pasteurella multocida*, is a highly contagious disease of poultry that can result in sudden death and high flock mortality, thereby disrupting food supply chains and causing major economic losses [43]. Similarly, swine dysentery caused by *Brachyspira hyodysenteriae* leads to severe diarrhea, reduced weight gain, and increased veterinary intervention costs [44, 45]. The economic burden associated with infectious bacterial diseases is considerable and includes both direct and indirect costs. In 2015, global livestock diseases were estimated to cause approximately $20 billion in annual losses due to decreased productivity, increased veterinary expenditures, and culling of infected animals [46]. Direct costs include veterinary care for infected animals, medications, diagnostic testing, and the implementation of biosecurity measures aimed at preventing disease spread. Indirect costs encompass losses associated with reduced productivity (e.g., decreased milk yield or weight gain), increased labor requirements during outbreak management, and potential losses resulting from trade restrictions and reduced market access [47, 48]. Food safety concerns further amplify these impacts. Contaminated animal‐derived products can serve as vehicles for zoonotic pathogens such as *Salmonella*, *Campylobacter*, and *Escherichia coli*, which are among the leading causes of foodborne illness worldwide [49]. According to the Centers for Disease Control and Prevention (CDC), foodborne pathogens are responsible for approximately 48 million illnesses annually in the United States alone. These infections impose a substantial burden on healthcare systems, with estimated annual costs of approximately $15.6 billion when accounting for medical expenses and productivity losses [50, 51]. The management of these infections often requires extensive veterinary care, including diagnostic testing, antimicrobial treatment, and, in severe cases, hospitalization. These interventions can become particularly costly during large‐scale outbreaks. Rapid and accurate identification of infectious agents is therefore critical for effective disease management. However, diagnostic procedures can be time‐consuming and resource‐intensive [52].

### 3.2. Antibiotic Resistance as a Critical Challenge Among Identifiable Foodborne and Zoonotic Bacteria

AMR is a major and growing concern in both veterinary and human health sectors. The widespread use and misuse of antibiotics in livestock production have contributed significantly to the emergence and dissemination of resistant bacterial strains. Common foodborne and zoonotic pathogens, including *Escherichia coli*, *Salmonella*, and *Staphylococcus aureus*, have increasingly demonstrated resistance to commonly used antibiotics, thereby complicating treatment strategies. Resistance can arise through multiple mechanisms, including spontaneous genetic mutations and the acquisition of resistance genes via horizontal gene transfer. As a result, a substantial proportion of bacterial isolates from animal and clinical sources exhibit resistance to one or more antimicrobial agents. For instance, studies have reported that more than 60% of *E. coli* isolates from livestock are resistant to at least one antibiotic [12–14, 53, 54]. The clinical and economic consequences of AMR are significant. Resistant infections often require more expensive or less effective therapeutic options and are associated with prolonged disease duration, increased risk of complications such as sepsis, and higher mortality rates [55, 56]. Furthermore, the transmission of antimicrobial‐resistant bacteria from animals to humans represents a critical public health challenge, complicating infection control and increasing the burden on healthcare systems [57–62]. In the United States alone, AMR is estimated to result in approximately $20 billion annually in excess healthcare costs and productivity losses [63]. Ribotyping is primarily used as an epidemiological typing tool that enables the identification and differentiation of bacterial strains across different sources. It has been widely applied in epidemiological surveillance to support source attribution and transmission analysis. Although it does not provide direct information on AMR determinants, the strain‐level data obtained through ribotyping can assist in understanding the dissemination of bacterial populations in food production and veterinary settings. Therefore, early and reliable identification of infectious bacteria using ribotyping may contribute to limiting the spread of resistant strains, particularly in food production and veterinary settings [64–66].

### 3.3. Transmission to Humans

The transmission of infectious bacteria from animals to humans occurs through multiple pathways, reflecting the complexity of zoonotic disease dynamics. These transmission routes include direct contact, foodborne exposure, environmental pathways, vector‐borne transmission, and airborne dissemination. Direct contact with infected animals or their bodily fluids can result in zoonotic infections, particularly among high‐risk occupational groups such as farmers, veterinarians, and animal handlers [67]. Foodborne transmission remains one of the most significant routes, occurring through the consumption of contaminated animal products, including undercooked meat and unpasteurized dairy products. The implementation of appropriate food handling practices and adequate cooking procedures is essential to reduce the risk of infection [68]. Environmental exposure also plays an important role. Contaminated water sources and soil may act as reservoirs for pathogens such as *Escherichia coli* and *Salmonella*, often as a result of agricultural runoff from infected livestock operations. These environmental pathways can contribute to increased incidence of foodborne disease and place additional strain on public health systems [69, 70]. Vector‐borne transmission involves the spread of bacterial pathogens through arthropods such as ticks. Pathogens including *Borrelia burgdorferi* and *Anaplasma phagocytophilum* rely on animal reservoirs to complete their life cycles and can infect both animals and humans [71]. Airborne transmission has also been reported in livestock production environments, where microorganisms may be associated with dust particles and dispersed through the air. Bacteria commonly detected in such environments include members of the genera *Streptococcus*, *Staphylococcus*, *Bacillus*, and *Clostridium*, as well as representatives of the family Enterobacteriaceae [72, 73].

### 3.4. Global Impact

The impact of infectious bacteria in animal‐derived foods is a global issue, although its magnitude and management vary across regions. Developed countries typically benefit from advanced surveillance systems, well‐established regulatory frameworks, and stricter control of antibiotic use in agriculture. These factors facilitate early detection of outbreaks and enable rapid implementation of control measures [74–76]. In contrast, developing countries often face challenges such as limited access to veterinary services, inadequate diagnostic infrastructure, and higher levels of antibiotic misuse in livestock production. These limitations can delay diagnosis and reduce the effectiveness of disease control strategies [76, 77]. Improved hygiene and biosecurity measures play a critical role in mitigating these risks. Farm‐level interventions, including proper sanitation, controlled animal movement, and routine health monitoring, are essential for reducing disease transmission among livestock [78, 79]. These challenges highlight the importance of reliable bacterial typing methods such as ribotyping for accurate identification and source tracking

## 4. Ribotyping for Identifying Infectious Bacteria in Animal Samples and Animal‐Based Foods

Although ribotyping has been employed as a molecular method in various studies, its application for identifying infectious bacteria in animal‐derived foods, animal products, and veterinary samples remains relatively limited compared with more recent genomic approaches (Table 1). In 2022, a study applied ribotyping based on the amplification of 16S and 23S rRNA genes using separate PCR assays to assess the prevalence of *Clostridioides difficile* in 250 raw bovine and ovine meat samples collected in Iran. The results showed that 6% of the samples were contaminated, with the highest prevalence observed in bovine meat (8%). Among the detected toxigenic genes, *tcdA* (33.33%) and *cdtA* (20%) were the most frequently identified, indicating the presence of potentially pathogenic strains in these food products [80]. Although this study demonstrates the applicability of ribotyping in food safety surveillance, it also highlights the dependence of detection outcomes on sample type and bacterial load, which may influence strain recovery and downstream typing success. Another study demonstrated that veterinary students may act as major carriers for the transmission of *C. difficile* spores within veterinary teaching clinics and hospital environments. This study evaluated contamination on the shoe soles of veterinarians and students and reported a high positivity rate of 95%. A total of 17 different PCR ribotypes were identified, with ribotype 010 being the most prevalent. These findings highlight the potential role of veterinary personnel in the dissemination of *C. difficile* in clinical settings [81]. This finding not only suggests potential occupational transmission but also reflects the high discriminatory capacity of PCR‐ribotyping in detecting strain diversity in high‐exposure environments. However, inter‐study comparability of ribotype distribution may be influenced by differences in primer sets, PCR conditions, and interpretation criteria, which limits direct cross‐laboratory comparisons. Wiedmann et al. investigated the ribotype diversity of *Listeria monocytogenes* strains associated with listeriosis outbreaks in ruminants. The study involved ribotyping isolates obtained from both clinical samples and silage consumed by affected animals in order to establish a causal link between feed and disease. In all but one outbreak, *L. monocytogenes* strains with identical ribotypes were successfully isolated from both clinical and silage samples, supporting the hypothesis of feed‐associated transmission. These findings underscore the value of ribotyping in epidemiological investigations aimed at tracing infection sources [82]. This study provides strong epidemiological evidence supporting feed‐associated transmission; however, the reproducibility of ribotype assignments across laboratories may still vary due to differences in restriction enzymes and hybridization conditions in classical ribotyping workflows. A large‐scale study conducted across 13 European countries examined the distribution of *C. difficile* ribotypes and sequence types among humans, animals, and food sources. Among the 106 ribotypes identified, 10 were shared across all three sources, whereas 68 were specific to individual sources. Certain ribotypes were exclusively found at the interface between human and food sources (RT023 and RT027) or between human and animal sources (RT009, RT045, and RT046). Overall, the study demonstrated substantial diversity in *C. difficile* ribotypes across Europe and suggested that specific ribotypes may be associated with distinct transmission pathways involving food and animals [83]. While this supports zoonotic and foodborne transmission pathways, the presence of source‐specific ribotypes also indicates significant genetic heterogeneity, which may not always be fully resolved by ribotyping alone. Another study evaluated *C. difficile* isolates from domestic animals and humans using PCR ribotyping, alongside PCR detection of toxin‐encoding genes. A total of 133 isolates were analyzed, including 92 from dogs, 21 from horses, 20 from humans, and one isolate each from a cat and a calf. Overall, 23 distinct ribotypes were identified: nine in dogs, 12 in horses, seven in humans, and one in each of the cat and calf samples. In dogs, humans, and horses, one or two ribotypes were predominant. Notably, 25% of the human isolates were indistinguishable from those identified in one or more animal species, indicating potential interspecies transmission. Toxin genes *tcdA* and *tcdB* were detected in all human, equine, and bovine isolates, as well as in 69% of canine isolates. These findings highlight both the genetic diversity of *C. difficile* and the potential for cross‐species transmission [84]. These findings reinforce the zoonotic potential of *C. difficile*, although variation in ribotype distribution across species may partly reflect methodological variability and limited resolution in distinguishing closely related strains. Ribotyping has also been used to characterize *Bordetella bronchiseptica* strains isolated from 11 host species worldwide, including pig, dog, rabbit, cat, guinea pig, horse, koala, human, turkey, rat, and leopard. Using the restriction enzyme PvuII, 16 distinct ribotypes were identified, each consisting of five to seven restriction fragments ranging from 1.8 to 5.6 kb. Approximately 88% of swine isolates were classified as ribotype 3, while 74.1% of canine isolates were identified as ribotype 4. In contrast, isolates from the remaining nine host species exhibited greater diversity, representing 15 different ribotypes. No correlation was observed between ribotype and geographic location. These results suggest that ribotyping can provide insights into host‐associated distribution patterns and transmission dynamics [85]. Avery et al. combined two genetic fingerprinting methods—pulsed‐field gel electrophoresis (PFGE) and ribotyping—to characterize 207 *E*. *coli* O157 isolates obtained from food animals, retail meats, and human clinical cases. PFGE was performed using the restriction endonuclease XbaI, whereas ribotyping involved simultaneous digestion of genomic DNA with PstI and SphI. The analysis generated 97 PFGE profiles and 51 ribotypes. Both methods demonstrated comparable discriminatory ability for the 154 epidemiologically unrelated isolates included in the study. Importantly, no correlation was observed between isolate source and specific PFGE or ribotype profiles. When combined, the two methods produced 146 distinct types, significantly increasing discriminatory power. This enhanced resolution allowed the identification of clonal groups within ribotype clusters that differed by only one or two PFGE bands, relationships that would not have been detected using a single method [86]. These findings highlight an important limitation of ribotyping alone, namely, its reduced resolution compared with combined or genome‐based typing approaches. Automated ribotyping using the RiboPrinter microbial characterization system has also been applied for molecular subtyping of *E. coli* O157:H7 in frozen beef patties. The highest number of culture‐confirmed positive results was detected using PCR, with most positive samples occurring within a specific processing time segment. Ribotyping classified isolates into two distinct pattern types corresponding to ribogroup A (50 isolates) and ribogroup B (3 isolates). The findings suggested that contamination originated from a specific batch of raw material introduced shortly before the 1725–1844 processing period. These results demonstrate the usefulness of ribotyping in identifying contamination sources in food production systems [87]. This phenomenon can be attributed to several methodological and biological factors, including incomplete restriction enzyme digestion, absence of restriction sites in target regions, or genetic variability within rRNA operons. In addition, the presence of multiple heterogeneous rRNA operon copies within bacterial genomes may generate atypical banding patterns that do not match reference databases, leading to classification failure. Such limitations directly affect the overall robustness and completeness of ribotyping‐based surveillance. A survey investigating *C. difficile* contamination in raw meat and carcass surface swab samples reported an overall prevalence of 3.71%, with detection rates of 2.91% in raw meat samples and 4.48% in carcass swabs. The highest prevalence was observed in raw sheep meat (5%) and sheep carcass swabs (7.50%). The study also documented substantial AMR, with the highest resistance rates observed for tetracycline (77.77%), erythromycin (72.22%), metronidazole (44.44%), clindamycin (38.88%), and ciprofloxacin (38.88%), whereas lower resistance was observed for meropenem (16.66%) and chloramphenicol (16.66%). Toxigenic genes *tcdA*, *tcdB*, *cdtA*, and *cdtB* were detected in 22.22%, 44.44%, and 16.66% of isolates, respectively, with the *tcdB + tcdA* profile being the most prevalent (27.77%). Ribotypes 027 and 078 were identified among the isolates. These findings indicate that food animals can act as reservoirs of toxigenic and antibiotic‐resistant *C. difficile*, with contamination likely occurring during slaughterhouse processing [88]. Another study compared four molecular typing methods—PFGE, random amplified polymorphic DNA (RAPD), ribotyping, and PCR‐ribotyping—for subtyping *Salmonella Enteritidis* isolates from an industrial poultry production chain. Antibiotic resistance rates were highest for enrofloxacin (18.5%), followed by furazolidone (15.7%), cefoxitin (1.8%), and both ciprofloxacin and ampicillin (0.9% each), while 6.4% of isolates were pan‐susceptible. All four typing methods produced similar results, indicating high genetic similarity (≥ 90%) among isolates, suggesting dissemination of closely related strains throughout the production chain. However, ribotyping based on Southern blot hybridization successfully typed 78.7% of isolates, whereas 21.3% remained untypeable despite repeated analysis using PstI. The typed isolates were classified into five profiles (R1–R4 and untypeable), indicating some limitations in typeability depending on methodological conditions [89]. These limitations are particularly evident in complex food matrices, where mixed populations, DNA quality, and methodological differences between laboratories may further reduce reproducibility. Therefore, these studies demonstrate that ribotyping can contribute to the identification and differentiation of bacterial strains in animal‐derived foods and veterinary samples. While its resolution is lower than that of genome‐based approaches, it remains a useful tool for epidemiological investigations, particularly for tracking transmission pathways and comparing strains across different sources.

**TABLE 1:** A summary of significant studies on various ribotyping techniques used to identify bacterial strains responsible for infections in animals, as well as those affecting humans, animal‐based foods, and veterinary samples.

Authors	Year	Bacteria	Source of sampling	Objective	Methodology	Key findings	Reference
Shahreza et al.	2022	*C. difficile*	Raw bovine and ovine meat samples	To assess the prevalence, toxigenic gene profile, and ribotyping of *C. difficile* strains	PCR‐based ribotyping targeting 16S and 23S rRNA genes	6% contamination overall; highest prevalence in bovine meat (8%); toxigenic genes tcdA (33.33%) and cdtA (20%) detected	[80]
Wojtacka et al.	2022	*C. difficile*	Shoe soles of veterinarians and students	This study was to assess the prevalence of *C. difficile* on shoe soles of veterinarians, veterinary support staff, and veterinary students	PCR ribotyping	95% positivity; 17 ribotypes identified; ribotype 010 most prevalent; veterinary students identified as potential transmission vectors	[81]
Wiedmann et al.	1996	*L. monocytogenes*	Ruminants and silage	To investigate ribotype diversity and link infection sources	Ribotyping of isolates from clinical samples and feed (silage)	Identical ribotypes found in clinical and silage samples in most outbreaks, indicating feed‐associated transmission	[82]
Rupnik et al.	2024	*C. difficile*	Humans, animals, and food	To assess distribution of ribotypes across hosts and food sources	PCR ribotyping and sequence typing	106 ribotypes identified; 10 shared across all sources; evidence of food‐ and animal‐associated transmission routes	[83]
Arroyo et al.	2005	*C. difficile*	Domestic animals and humans	To compare ribotypes and assess interspecies transmission	PCR ribotyping	23 ribotypes identified; overlap between human and animal isolates (25% indistinguishable strains)	[84]
Register et al.	1997	*B. bronchiseptica*	Multiple animal species (pig, dog, rabbit, etc.)	To characterize genetic diversity across host species	Ribotyping using restriction enzyme PvuII	16 ribotypes identified; no clear correlation between geography and ribotype	[85]
Avery et al.	2002	*E. coli* O157	Food animals, retail meats, humans	To compare PFGE and ribotyping for strain discrimination	PFGE and ribotyping combined approach	146 distinct types identified; high discriminatory power when combining methods	[86]
Pruett et al.	2002	*E. coli* O157:H7	Frozen beef patties	To detect and subtype contamination in beef products	PCR and ribotyping	Two ribogroups identified (A and B); confirmed usefulness for detecting low‐level contamination	[87]
Bacheno et al.	2022	*C. difficile*	Raw meat and carcass swabs	To evaluate prevalence and antimicrobial resistance	PCR ribotyping with toxin gene detection	3.71% prevalence; presence of ribotypes 027 and 078; carcasses identified as reservoirs of toxigenic strains	[88]
Monte et al.	2020	*Salmonella Enteritidis*	Poultry production chain	To evaluate strain diversity and typing performance	Ribotyping and comparative molecular typing	78.7% typeability; 5 ribotype profiles identified; some isolates remained untypeable	[89]

Abbreviations: *B. bronchiseptica*, *Bordetella bronchiseptica*; *C. difficile*, *Clostridioides difficile*; *E. coli*, *Escherichia coli*; *L. monocytogenes*, *Listeria monocytogenes*; PCR, polymerase chain reaction; PFGE, pulsed‐field gel electrophoresis; rRNA, ribosomal RNA.

## 5. Comparison of Ribotyping With Current Bacterial Typing Methods

Recent advances in molecular epidemiology have substantially reshaped bacterial typing strategies, leading to a progressive shift from classical approaches such as ribotyping toward high‐resolution genome‐based methods. Although ribotyping has historically contributed to the identification and epidemiological tracking of bacterial pathogens, its role has diminished in the context of modern genomic technologies that provide significantly higher resolution and analytical depth. This transition is largely driven by the increasing accessibility, affordability, and analytical power of next‐generation sequencing platforms, which enable comprehensive characterization of bacterial genomes. Recent advances in genomic epidemiology have been demonstrated through applied studies utilizing WGS in animal‐derived food systems and veterinary contexts. For example, a study conducted in Italy performed WGS on 257 *Campylobacter* isolates obtained from both human clinical samples and chicken‐derived food products. The analysis revealed substantial genetic diversity and identified multiple genetic clusters linking human infections to poultry sources, demonstrating the utility of WGS for high‐resolution characterization and source attribution of foodborne pathogens across the animal–human interface [90]. Similarly, a multicountry study published in 2024 applied WGS and metagenomic approaches to characterize enteric pathogens, including *Salmonella, E*. *coli,* and *Campylobacter*, across human, animal, and environmental sources. By analyzing hundreds of isolates collected between 2019 and 2023, the study demonstrated that WGS enables detailed genomic characterization and tracking of pathogen distribution across interconnected reservoirs, supporting integrated surveillance within a One Health framework [91]. WGS is now widely regarded as the gold standard for bacterial typing due to its unparalleled discriminatory power and ability to resolve genetic relationships at the single‐nucleotide level. Unlike ribotyping, which targets conserved rRNA gene regions, WGS captures the entire genomic content of an organism, allowing detailed analysis of virulence determinants, AMR genes, and evolutionary dynamics. Several studies have demonstrated that WGS enables precise outbreak tracking and source attribution, particularly in foodborne pathogens, and supports real‐time epidemiological surveillance. Furthermore, WGS has increasingly been implemented in public health laboratories as a comprehensive tool capable of simultaneous species identification, resistance profiling, and phylogenetic analysis [92–96].

Multilocus sequence typing (MLST) represents an established sequence‐based approach that analyzes variation in a set of conserved housekeeping genes. Compared with ribotyping, MLST provides improved reproducibility, portability, and inter‐laboratory comparability through standardized allele‐based databases. It has been widely used for population structure analysis and long‐term epidemiological studies across diverse bacterial species. However, MLST examines only a limited portion of the genome and often lacks sufficient discriminatory power for resolving closely related strains during outbreak investigations, particularly when compared with genome‐wide approaches [97–99].

Single‐nucleotide polymorphism (SNP)–based approaches have emerged as high‐resolution tools for bacterial typing, enabling precise discrimination among closely related isolates through the identification of single‐base variations distributed across the genome. These approaches facilitate fine‐scale phylogenetic reconstruction and provide robust frameworks for tracking transmission pathways and investigating outbreak dynamics in both clinical and foodborne settings. Recent studies have demonstrated that SNP‐based typing allows the assignment of isolates into hierarchical clusters reflecting genetic relatedness, thereby supporting epidemiological linkage and real‐time surveillance applications. Moreover, SNP‐based analyses offer enhanced sensitivity for detecting microevolutionary changes within bacterial populations, which is critical for understanding pathogen adaptation and dissemination. However, these approaches are not without limitations, as their accuracy may be affected by recombination events that introduce multiple nucleotide changes simultaneously, potentially confounding phylogenetic inference. In addition, SNP‐based workflows require standardized analytical pipelines and substantial computational resources, which may limit their implementation in routine laboratory settings [100–102].

In contrast, ribotyping is inherently limited by its focus on rRNA gene regions, which do not capture the full extent of genomic diversity within bacterial populations. Consequently, its discriminatory power is often insufficient for distinguishing closely related strains, especially in outbreak scenarios where high‐resolution typing is essential. Additionally, variability in laboratory protocols, including differences in restriction enzymes and analytical methods, may affect reproducibility and comparability across studies, further limiting its application in modern surveillance frameworks [103, 104].

Despite these limitations, ribotyping retains certain practical advantages. It is relatively cost‐effective, technically less demanding, and does not require advanced sequencing infrastructure or bioinformatics expertise. These characteristics make it suitable for use in resource‐limited settings or as a preliminary screening tool. Moreover, historical ribotyping datasets remain valuable for longitudinal comparisons, particularly in studies aiming to maintain consistency with earlier epidemiological data, as the method has been widely used in earlier molecular epidemiology investigations [24, 104].

Therefore, while ribotyping presents certain inherent limitations, particularly in terms of discriminatory power and resolution compared with high‐resolution molecular approaches, it continues to play a meaningful role in bacterial typing. Its relative simplicity, cost‐effectiveness, and minimal requirement for advanced sequencing infrastructure make it a practical and accessible tool, especially in routine laboratory settings and in regions with limited resources. In many applied contexts, including veterinary diagnostics and food safety monitoring, ribotyping remains a useful method for preliminary identification, epidemiological screening, and comparison with historical datasets. Therefore, despite the emergence of more advanced genomic techniques, ribotyping retains its relevance as a complementary and, in some cases, preferred approach where rapid, affordable, and standardized bacterial identification is required. Integrating ribotyping with newer molecular methods may further enhance its utility within modern surveillance frameworks.

## 6. Conclusion

The studies reviewed demonstrate that ribotyping remains a useful method for identifying infectious bacteria in animal‐derived foods and veterinary samples, particularly as a cost‐effective and accessible tool in routine laboratory settings. However, infectious bacteria continue to pose complex challenges affecting animal health, food safety, public health, and economic stability worldwide. The increasing prevalence of antibiotic‐resistant strains further emphasizes the need for reliable and timely identification methods to support effective control strategies and limit transmission across the animal–human interface. Despite its practical advantages, ribotyping has inherent limitations in discriminatory power compared to high‐resolution genomic approaches. Therefore, future research should focus on improving the applicability and relevance of ribotyping within modern surveillance frameworks. In particular, the standardization of ribotyping protocols is essential to enhance reproducibility and facilitate its use in regulatory food safety systems. In addition, integrating ribotyping with WGS approaches may provide a balanced strategy, especially in low‐resource settings where full genomic analysis may not always be feasible. The development of open‐access ribotype databases covering animal, food, and human isolates would further support data sharing and strengthen global surveillance efforts within a One Health framework. Therefore, while ribotyping alone may not meet the demands of high‐resolution epidemiological investigations, it continues to play a complementary role in bacterial identification and surveillance. Strengthening its integration with emerging genomic technologies and improving methodological standardization will enhance its utility in addressing current and future challenges related to infectious bacteria in animal‐derived food systems. Addressing these challenges requires coordinated efforts across various sectors—including veterinary medicine, microbiology, agriculture, healthcare, and public policy—to implement effective surveillance systems, promote responsible antibiotic use, enhance hygiene practices, and educate stakeholders about the risks associated with infectious diseases in animals. By understanding the complexities surrounding infectious bacteria in animal populations and their implications for human health, stakeholders can work collaboratively toward developing strategies that mitigate risks while promoting animal welfare and public safety.

## Author Contributions

Zahra Pourramezan Seyedi, Baharak Mirzavand, and Mohammad Taghi Ashoobi: writing and editing original draft. Majid Taati Moghadam: conceptualization, writing–original draft, and project administration. Jamile Nowsani: investigation and editing original draft.

## Ethics Statement

The authors have nothing to report.

## Conflicts of Interest

The authors declare no conflicts of interest.

## Data Availability

The data that support the findings of this study are available on request from the corresponding authors. The data are not publicly available due to privacy or ethical restrictions.
